# Correction: Seismic response of a mid-story isolated stilted structure in mountainous areas

**DOI:** 10.1371/journal.pone.0315954

**Published:** 2024-12-12

**Authors:** Jianhua Li, Dewen Liu

The second author, Dewen Liu, is incorrectly noted as the corresponding author. The correct corresponding author is Jianhua Li. Jianhua Li’s email address is: ljhgood999@163.com.

The funding statement for this paper is incorrect. The correct Funding statement is as follows: This work was supported by a grant under the project ‘Reform Practice and Research of “Three Education” Based on the Courses of Construction Data Management’. The Courses of Construction Data Management‘ (No. WZYGJzd202005) and’ Digital Management Technical Service for Industrial Base B2-1 Plot Project in Xianyan Township’ (No. H2023224) from Wenzhou Polytechnic, China.
