# Supplementary material for: Seismic response of a mid-story isolated stilted structure in mountainous areas
Source: PLoS One. 2024 Oct 29;19(10):e0312503. doi: 10.1371/journal.pone.0312503 (PMC11521315; doi:10.1371/journal.pone.0312503)
Supplement: Attachment — Submitted filename: PONE-D-24-16917.docx [file pone.0312503.s001.docx]

Based on the engineering case of the mid-story isolated stilted structures in Chongqing, the effects of considering SSI and not considering SSI on the seismic response of the mid-story isolated stilted structures in mountainous terrain were studied in this paper, and the effects of different mountain slopes on the seismic response of the mid-story isolated stilted structures with and without considering SSI were analyzed. In addition, the elastic-plastic time-history analysis of the mid-story isolated stilted structures under earthquakes in the down and transverse slopes was also conducted.

Comments:

1. Please check the manuscript for grammatical errors.

2. Please add the basis for selecting seismic motions in Section 2.3.

3. Please explain the meaning of "down slope" and "transverse slope", and explain the main differences between them in the manuscript.

4. In Table 1, the cross-sectional dimensions in mm of the frame columns are 600x600 and of main beams 700x350 and of the secondary beams 600×300. Usually, the first number is the width and the second is the depth of the member. Is the first number here width or depth? Please clarify.

5. In Section 4.2, "and the inter-story shear of six different mid-story isolated stilted structures as shown in Fig. 5 and Fig. 6" should be changed to "and the inter-story shear of six different mid-story isolated stilted structures as shown in Fig. 5 and Fig. 6". There are many similar errors in the manuscript. Please check and modify them.

6. The legends in Figures 4, 5, 6, 7, 8 and 11 are too small to see clearly. Please improve them.
