# Supplementary material for: Seismic response of a mid-story isolated stilted structure in mountainous areas
Source: PLoS One. 2024 Oct 29;19(10):e0312503. doi: 10.1371/journal.pone.0312503 (PMC11521315; doi:10.1371/journal.pone.0312503)
Supplement: Attachment — Submitted filename: Response to Reviewers.docx [file pone.0312503.s003.docx]

**Response to reviewers**

Dear Editor,

Thanks for your letter and thanks for the review comments concerning our manuscript entitled “Seismic Response of a Mid-story Isolated Stilted Structure in Mountainous Areas”. The comments are all valuable and helpful for revising and improving our paper. We have studied all comments carefully and have made conscientious corrections. Modified portions are marked in red on the paper. The main corrections to the paper and the responses to the review comments are listed as follows:

Below are our detailed responses to all the comments.

**Reply to** **Academic Editor**

Dear Professor,

Thank you for giving us the opportunity to revise our paper. We have read your comments carefully and revised our paper. Thank you very much for your valuable comments, which gave me a better understanding of this research.

We have referred to Fig. 3 in the text.

**Comment 1:** The manuscript written English and fashion does not meet the standard of PLOS ONE. Please consult professional proofreading services to improve the English of the manuscript.

**Response 1:** We appreciate the valuable comments and suggestions provided by the academic editor. These comments have played a constructive role in refining the content and structure of the paper. We have improved the English.

**Comment 2:** There are several editorial mistakes, please correct them.

**Response 2:** Thank you very much for your valuable comments. We have checked and corrected the discovered editorial mistakes.

**Comment 3:** Please increase the legend size of the figures. It is hard to read them.

**Response 3:** Thank you very much for your valuable comments. We have increased the size of the legends for figures 5, 6, 7, 8(a), 9(b), 10, and 12.

**Comment 4:** Please highlight the design response spectra in Figure 4.

**Response 4:** Thank you very much for your valuable comments. We have modified figure 5 to highlight the design response spectra.

**Comment 5:** Please discuss the results of base shear.

**Response 5:** Thank you very much for your valuable comments. In Section 4.2, we have added a discussion on base shear. Content as follows：

“The base shear and inter-story shear are influenced by the slope to a similar extent. When considering the SSI effect, the base shear shows a noticeable increase with the increase in the slope of the mountainous terrain. However, when not considering the SSI effect, an increase in the slope of the mountainous terrain does not lead to a significant increase in base shear. This indicates that when considering the SSI in mountainous areas, the slope soil cannot be regarded as a rigid body. As the slope increases, the volume of soil under the structure also increases, and the mutual propagation and exchange effect of vibration energy increases.”

**Comment 6:** Please improve the conclusions and write down them point-wise for ease of readers.

**Response 6:** Thank you very much for your valuable comments. We have improved the conclusions and written them down point-wise.

Thank you again for your valuable comments. I am looking forward to hearing from you soon.

Yours Sincerely,

Dewen Liu
